# The Immediate as Distinguished from the Permanent Treatment of Disease

**Published:** 1879-12

**Authors:** 


The Immediate as Distinguished from the Permanent Treat ment of Disease.—Dr. J. Milner Fothergill recently read an instructive paper on this subject, before the Harveian Society of London. He pointed out how in many cases the treatment which gives immediate relief is not that to be continued in the permanent interests of the patient. He instanced first the free use of opium in the hacking cough of phthisis, and in chronic bronchitis, which gave immediate relief, but did harm eventually. Then, in the diarrhœa due to impacted masses in the rectum, astringent mixtures might give immediate relieve, but they were not curative, while removal of the masses was. So, too, in neuralgia, the injection of morphia eased the pain for the time, but if continued, was more likely to confirm it than cure it. Likewise in dyspepsia, of reflex origin ; it was all very well to give the ordinary mixture to relieve it, but its cure depended upon the removal of the exciting cause. In gout, too, the application of cold, or of leeches, gives instant relief ; but he quoted Garrod in illustration of the evil consequences which follow such treatment. But of all instances of the conflict between the present and the permanent treatment of disease, that furnished by endocarditis, was, he said, the most striking. It was the rule to give tonics as soon as possible, and to get the patient up ; but, he contended, the proper plan of treatment is to keep the patient flat in bed for some days after all evidence of active mischief has passed away. The growth of connective tissue in the valve curtains, which is lighted up by the inflammatory storm that passes over the endocardium, persists some time after the endocarditis itself is over ; and it is the mutilation caused by the contraction of the neoplasm which we have chiefly to dread. Consequently, the true line of practice is to reduce the strain upon the inflamed valve curtains by complete rest, and the administration of agents which lower the blood-pressure within the heart and arteries. The more the connective tissue growth could be limited at the outset, the less the future mutilation of the valves. A few days in bed are nothing, compared to future valvular disease.

